# Supplementary material for: Proteome and phospholipidome interrelationship of synovial fluid-derived extracellular vesicles in equine osteoarthritis: An exploratory ‘multi-omics’ study to identify composite biomarkers
Source: Biochem Biophys Rep. 2024 Jan 18;37:101635. doi: 10.1016/j.bbrep.2023.101635 (PMC10828605; doi:10.1016/j.bbrep.2023.101635)
Supplement: Multimedia component 1 [file mmc1.pdf]

Supplementary materials and methods

Diagnostic criteria and classification of collected samples

OA was diagnosed based on clinical examination, including subjective lameness scoring according to the American Association of Equine Practitioners (AAEP) lameness scale, radiographic imaging and diagnostic analgesia. Classification as "mild OA" – a mild form of the disease (minor lesions, e.g. small osteophytes and limited subchondral bone sclerosis) and "severe OA" – a severe form of the disease (bone deformation, clear subchondral bone sclerosis, narrowing of joint space and formation of larger osteophytes) was based on radiography; radiographic examples of each OA phenotype are shown in Suppl. Table 1.

Table 1: Radiographic criteria used for the classification of the healthy, mild, and severe OA phenotypes. Arrows indicate osteophyte formation and subchondral osteolysis, and R and L denote right or left limb.

Group	Description	Radiograph
Healthy	No lesions	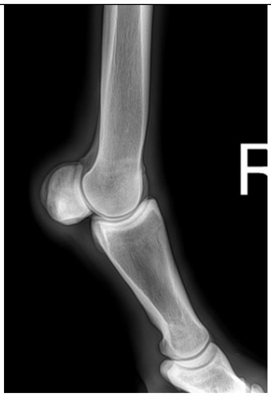
Mild OA	Small osteophyte and subchondral osteolysis	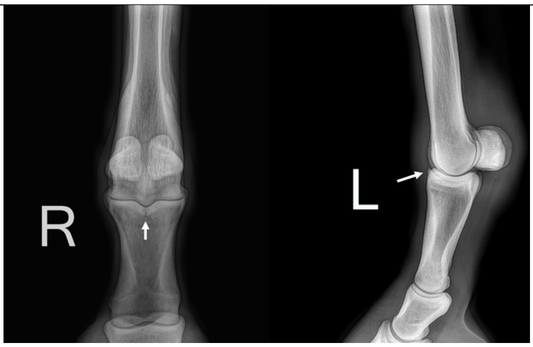
Severe OA	Bone deformation, narrowing of joint space, subchondral bone sclerosis, osteophytes	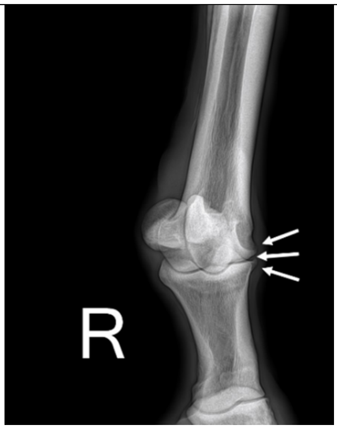

Extracellular vesicle isolation

Differential centrifugation

EVs from SF were isolated using a published and validated method [1]. First, the pooled cell-free SF samples (5 mL) were incubated at 37 °C for 15 minutes with HYase (5 mg/mL; HYase type II from sheep testes, Sigma- Aldrich, St. Louis, MO, USA) while vortexing every 5 minutes. Next, (protein) aggregates and debris were removed by centrifuging at 1,000 × *g* for 10 minutes at RT (Avanti J-15R; Beckman Coulter Inc., Brea, CA, USA). Next, the supernatants were transferred into SW40 tubes (Beckman Coulter Inc., Brea, CA, USA) and mixed with phosphate-buffered saline (PBS) to a volume of 12 mL, and centrifuged at 10,000 × *g* for 35 minutes (8,900 RPM; RCF average 10,003 × *g*; RCF max 14,088 × *g*; κ-Factor 2,771), followed by 100,000 × *g* for 65 minutes (28,000 RPM; RCF average 99,004 × *g*; RCF max 139,439 × *g*; κ-Factor 280). The 40 Ti Beckman Coulter rotors were used in an Optima™ L-90K or Optima™ XPN-80 ultracentrifuges. EV pellets were resuspended in 300 μL PBS+0.1% Bovine Serum Albumin (BSA) depleted of EVs (EV-depleted BSA) was achieved by overnight ultracentrifugation of 5% BSA at 4 °C at 32,000 RPM (RCF average 125,755 × g; RCF max 174,889 × g; κ-Factor 204).

Sucrose density gradient

EV pellets were thoroughly mixed with 1.2 mL 2.5 M sucrose solution (J.T. Baker; Phillipsburg, NJ, USA) in a new SW40 tube and overlaid with fourteen sucrose solutions of decreasing density (from 2 M to 0.4 M), creating a discontinuous sucrose gradient. Gradients were centrifuged at 200,000 × *g* for 16 hours at 4 °C in an SW40-Ti rotor (39,000 RPM; RCF average 192,072 × *g*; RCF max 270,519 × *g*; κ-factor 144.5). Twelve fractions of 1 mL were collected from the top (lowest density) to the bottom (highest density). Fraction densities were determined by refractometry. EV-containing fractions (validated in [1; 2]) were pooled (densities between 1.10-1.16 g/mL) and pipetted into SW32 ultracentrifuge tubes for lipidomics and SW60 for proteomics analysis. EVs were pelleted by centrifugation for 95 minutes at 120,000 × *g* at 4°C (SW32 Ti. 32,000 RPM; RCF average 127,755 × *g*; RCF max 174,899 × *g*; κ-factor 204, or SW60 Ti, 35,000 RPM; RCF average 125,812 × *g*; RCF max 165,052 × *g*; κ-Factor 133). Subsequently, EVs for lipidomics were resuspended in 100 μL of PBS. For proteomics, dried EV pellets were snap-frozen immediately at -20 °C for later analysis.
Relevant data regarding the experimental details for EV isolation and characterization have been submitted to the EV-TRACK knowledgebase (EV-TRACK ID: EV230607) [3].

Single-EV-based high-resolution flow cytometry

Labelling of EV pellets with PKH67

Generic fluorescent staining of EVs was performed with the PKH67 labeling kit (Sigma-Aldrich, St. Louis, USA) as previously described [2; 4], with minor modifications indicated below. EV pellets were resuspended in 20 μL PBS+0.1% BSA EV-depleted with 30 μL diluent C. Then, 50 μL of diluent C with 1.5 μL of PKH67 dye were added. The staining process was halted by adding 50 μL of EV-depleted RPMI/10% FBS (Roswell Park Memorial Institute/fetal bovine serum) after 3 minutes of incubation at room temperature. Next, the labeled EVs were combined with 2.5 M sucrose to continue the previously described density gradient ultracentrifugation process for EV separation. Throughout the whole PKH67 labeling and sucrose gradient ultracentrifugation process, a procedural control sample (20 μL PBS+0.1% BSA EV-depleted +30 μL diluent C, without sample EVs) was used as a control sample for high-resolution FCM.

Single EV-based high-resolution FCM analysis

An optimised BD Influx jet-in-air- flow cytometer (Becton Dickinson Biosciences, San Jose, CA, USA) fully tailored for single EV analysis was employed [4; 5]. In short, a workspace was loaded that had the optimised PMT parameters and pre-defined gates for the detection of 200 nm yellow-green (505/515) FluoSphere beads (Invitrogen, F8848). After the fluid stream and lasers were aligned, the 200 nm bead population had to fulfil the requirements of pre-defined mean fluorescent intensity and scatter values inside these gates while exhibiting the lowest coefficient of variation for side scatter, forward scatter, and FL-1 fluorescence. All measurements in this study used the same fluorescent threshold level, which was established by running a clean PBS sample and allowing an event rate of < 20 events/ second. All samples were run for a fixed period of 30 seconds. The EV concentration was calculated based on the number of fluorescent events detected and normalised for the flow rate of 12.8 μL/minute, dilution factor, and measurement time. The final EV concentration per mL of SF was determined for the EV-enriched sucrose fractions F7–F10 (densities 1.10 g/mL–1.16 g/mL), adjusted based on the SF starting volume. The procedural control sample revealed no noteworthy background events (<500 events/ 30 secs) in gradient fractions of interest (data not shown).
The BD FACS Software v1.0.1.654 (BD Biosciences, San Jose, CA, USA) was used to collect the data, and the FlowJo v10.07 software (FlowJo, Ashland, OR, USA) was used for analysis. The MIFlowCyt author checklist can be found on Supplementary Table 2, and the MIFlowCyt-EV framework on Supplementary Table 3 [6].

Lipidomic analysis

Lipid extraction

Lipids were extracted following the Bligh & Dyer method [7] with slight modifications. First, 0.7 mL of fresh deionized water was mixed with 100 μL of samples, 2 mL methanol, and 1 mL chloroform. The samples were incubated for 20 minutes, then 2 mL chloroform and 2 mL of deionized water were added, and the mixture was vortexed. The resultant hydrophilic and hydrophobic phases were separated by centrifugation at room temperature for 5 minutes at 2,000 $\times$ g. The hydrophobic bottom phase was transferred to a new conical glass tube. The extraction of the remaining sample (hydrophilic phase) was repeated with an additional 2 mL of chloroform to ensure that all lipids were collected. The samples were dried under nitrogen gas injection and stored in a nitrogen atmosphere at -20 °C. During the lipid extraction, one sample from healthy joints was lost. Therefore, n=6 SF-EV samples of the group with healthy joints were used for all lipidomics analyses and subsequent omics integration.

Mass spectrometry lipidomics

Dried lipid pellets were resuspended in 30 μL chloroform/methanol (1:1) and analyzed as described previously [2; 8]. A quality control sample composed of all samples in the same ratio together with the SPLASH® Lipidomix® Mass Spec Standard (Avanti Polar Lipids, Inc., Alabaster USA) was created for subsequent lipid quantification and added in the MS run. A hydrophilic interaction liquid chromatography (HILIC) column (2.6 μm Kinetex HILIC 100, 50 x 4.6 mm, Phenomenex, Torrance, USA) was loaded with 10 μL of lipid extract. Gradient elution on an Agilent 1290 InfinityII UPLC (Agilent, CA) separated the lipid classes. Solvent A consisted of acetonitrile/acetone (9:1) with 0.1% formic acid, and solvent B was composed of acetonitrile/H_2O (7:3) with 0.1% formic acid and 10 mM ammonium formate. The gradient profile was: minute 0 to 1: 100% A; minute 1 to 3: 50% A + 50% B; minute 3 to 5: 100% B, with a 1 mL/min flow rate. Without further re-equilibration of the column, samples were injected.
The samples were analyzed with a Fusion Orbitrap MS (ThermoFisher Scientific, Waltham, USA) via a heated electrospray ionization (HESI) source with the following parameters: negative ion spray voltage, 3.6 kV; aux sheath gas flow rate, 54Arb; gas flow rate, 7Arb; sweep gas flow rate, 1Arb; ion transfer tube temperature, 350°C; vaporizer temperature, 450°C; scan range, 350–950 m/z at a resolution of 120,000.

Lipid annotation

The msconvert ProteoWizard [9] was used to convert the RAW format to mzML with the "peakPicking filter vendor msLevel = 1-" parameter selected. The package XCMS version 3.10.2 [10] was used to perform liquid chromatography mass spectrometry (LC/MS) peak-picking, sample-grouping, and retention time correction on the mzML files running under R version 4.1.2. The identified LC/MS peaks (features) were annotated based on retention time, exact m/z-ratio, and, if present in at least 3 out of 13 pooled samples (Healthy n=6, OA n=4, Advanced OA n=3), MS peaks were annotated using an in-silico phospholipid database. The features were adjusted to account for 13 C and 37 Cl isotope overlap and verified based on isotope distribution and alternative adducts.For the analysis, only major adducts (i.e., $[M^{+}Cl^{-}]^{-}$ for HexnCer and $[M^{+}HCOO^{-}]^{-}$ for PC and SM, and $[M-H^{+}]^{-}$ for all other lipid classes) were used, while the minor ones were ignored to avoid under-quantification of less prominent lipid species. The RAW and mzML converted mass spectrometry data is deposited in the YODA repository of Utrecht University [11].

Proteomic analysis

Protein extraction

EV pellets were resuspended in 200 μL of urea lysis buffer (6 M Urea; Sigma-Aldrich, Dorset, United Kingdom), 1 M ammonium bicarbonate (Fluka Chemicals Ltd., Gillingham, UK) and 0.5% sodium deoxycholate (Sigma-Al-drich, Dorset, United Kingdom). Samples were sonicated at 5 μm for 3 $\times$ 10 seconds per sample, with 1-min rest on ice between each sonication round as previously described [12].

SDS PAGE & silver stain

Sodium dodecyl sulfate-polyacrylamide gel electrophoresis (SDS-PAGE) was used to separate proteins from EV protein extracts. 7.5 μL of 2x Novex™ Tris-Glycine SDS Sample Buffer (ThermoFisher Scientific, Paisley, UK), supplemented with 8% of 2-Mercaptoethanol (Sigma-Aldrich, Dorset, UK), was added to 7.5 μL of sample SF-EV protein lysate. Samples were mixed and heated at 100°C for 10 minutes to denature proteins, then placed on ice. Electrophoresis was performed using A NuPAGE™ 4 to 12%, Bis-Tris gel (ThermoFisher Scientific, Paisley, UK) and 1x NuPAGE® MES Running Buffer (ThermoFisher Scientific, Paisley, UK) (diluted from the 20x stock in ultrapure water). Samples were loaded onto the gel alongside the Novex™ Sharp Pre-stained Protein Standard ladder (ThermoFisher Scientific, Paisley, UK). Gels were run at 100V until completion of electrophoresis and visualized using silver stain (Thermofisher Scientific, Paisley, UK) according to the manufacturer's guidelines as previously done [12].

On bead digestion

Hydrophilic and hydrophobic magnetic beads were used for EV protein digestion in order to remove the urea lysis buffer that was not mass spectrometry compatible. Beads were suspended within the lysed EV samples in order for extracted EV proteins to bind to the surface of the bead, and thus, a tryptic digest was performed on bead.

Specifically, 95 μL of lysed and sonicated equine SF-EV were treated with 5 mM dithiothreitol (DTT) (Sigma-Aldrich, Dorset, UK) 100 mM at 60°C and shaken at 1000 rpm for 30 mins on an orbital shaker. Iodoacetamide (Sigma-Aldrich, Dorset, UK) was then added to a final concentration of 20 mM, and the samples were incubated at room temperature in the dark for 30 min. Following this, 5 mM DTT was added to each sample and incubated at room temperature for 15 min. Hydrophilic and hydrophobic magnetic carboxylate SpeedBeads (SP3 beads, total of 12 μL) (Cytiva, Massachusetts, US) were added to each sample, followed by 120 μL ethanol (Sigma- Aldrich, Dorset, UK). Samples were then incubated at 24°C and shaken at 1000 rpm for 1 h. The beads were separated from samples using a magnetic stand, washed thrice with 180 μL 80% ethanol and resuspended in 100 mM ammonium bicarbonate (Fluka Chemicals Ltd., Gillingham, UK, 4 μg). Trypsin/LysC (2.4 μg) (Promega) was added to each sample. Samples were placed in a sonicator bath and sonicated for 30 seconds to disaggregate the beads before being incubated overnight at 37°C and shaken at 1000 rpm. Beads were removed from the samples using the magnetic stand, and the supernatants were acidified by adding 1 μL 10% trifluoroacetic acid (Sigma- Aldrich, Dorset, UK). Samples were then desalted using an Agilent mRP-C18 column, dried in a SpeedVac and resuspended in 0.1% formic acid. The UV absorbance measured during desalting was used to normalize the loading for mass spectrometry analysis, with a final volume of 5 μL being loaded on the nano-LC column as previously described [12].

Data-dependent acquisition for the generation of an equine SF EV spectral library

Equine SF was pooled using samples from the metacarpophalangeal joint from our equine musculoskeletal biobank (VREC561) and samples collected in previous studies from the carpal and metacarpal joint of healthy horses as well as those with OA, resulting in a total of 11 ml SF. These samples were analyzed as previously described in order to generate the necessary reference library [13].

Data-independent acquisition proteomics (SWATH)

A data-independent proteomic approach was utilised in the form of Sequential Windowed Acquisition of all theoretical fragments (SWATH). Data were acquired using the same 2-hour gradient as the library fractions [12]. SWATH acquisitions were performed on a Triple TOF 6600 (Sciex) via an Eksigent nanoLC 415 fitted with an ACQUITY UPLC Peptide BEH C18 nanoACQUITY Column (Waters, UK) and a bioZEN 2.6 μm Peptide XB-C18 (FS) nano column (250 mm x 75 μm, Phenomenex). Data were acquired using 100 windows of variable effective isolation width to cover a precursor m/z range of 400-1500 and a product ion m/z range of 100-1650. Scan times were 50 ms for TOF-MS and 36 ms for each SWATH window, giving a total cycle time of 3.7 seconds. Retention time alignment and peptide/protein quantification were performed by Data-Independent Acquisition by Neural Networks (DIA-NN), using the same reference horse proteome as described previously to reannotate the library. A precursor FDR of 1%, with match between runs and unrelated runs was selected. The mass spectrometry proteomics data were deposited to the ProteomeXchange Consortium via PRIDE proteome exchange [12] (identifier PXD042765). Both proteomics and lipidomics datasets have been submitted to vesiclepedia [14].

Statistical analysis

EV characterization

Comparison in EV concentration (fluorescent events/mL) between healthy and OA was done by using a Student's t-test.

Proteomics

Statistical analysis of proteomics data was carried out using the R statistical programming environment or Metaboanalyst [15]. The data were quality controlled; proteins with complete observations were normalized using probabilistic quotient normalization (PQN) and log-transformed (base 10) for downstream analysis. Unsupervised multivariate analysis in the form of principal component analysis (PCA) was performed, along with heat map analysis using analysis of variance (ANOVA) and Pearson distance. One-way ANOVA with Tukey's post hoc test was attributed to statistically significant proteins in their respective group comparison. Following ANOVA, a fold change analysis was conducted.

Lipidomics and omics data integration

For lipidomics analysis, the data were normalized based on the sum of total lipids per pool sample – i.e., each lipid value in a pooled sample was divided by the total sum of lipids in the same pool sample and multiplied by 0.01; thus, the relative abundances sum up to 100. A minimum of three biological-pool replicates were used for statistical analyses.

Data analysis was run with R version 4.1.2 [16]. Pareto scaling was performed for the PCA, thus dividing each variable by the square root of its standard deviation. Heatmap and cluster analysis was performed on Spearman correlations with a set speed of two – among the 50 most abundant lipid species in all sample groups – using the R-package ComplexHeatmap v1.12.0 [17].

Data integration was performed with the R package mixOmics v6.12.2. [18] on lipidomic and proteomics data normalized by the sum (as described for lipidomics analysis) followed by R scaling and centering, which determines the vector's mean and standard deviation, deducts the mean from the vector and divides it by the standard deviation. An unsupervised sparse Partial Least Squares (a linear, multivariate regression method for data reduction to assess the relationship between independent and dependent variables) was used to integrate the datasets. The relevance network plot was set with a correlation cut-off of 0.7 to allow readability of the displayed proteins and phospholipids. Differences between the proposed proteins and phospholipid percentages for the composite biomarker were analyzed with the rank-based non-parametric Kruskal-Wallis test, followed by the multiple pairwise comparisons with Dunn's test. Significance was defined as p-value < 0.05. Statistical tests were done with GraphPad Prism 9.

Functional enrichment analysis

Functional enrichment analysis was performed on proteomic data using Ingenuity Pathway Analysis (IPA; Qiagen, Hilden, The Netherlands) in order to provide functional analyses, networks, canonical pathways, and related molecular and pathological functions by using protein p-values obtained through One-way ANOVA with Tukey's post hoc test, and associated log2 fold change, including those differentially expressed (values of less than 0.05), and associated log2 fold change. UniProt_Horse accession codes were used as protein identifiers, and the Qiagen Ingenuity Knowledge Base was used as a reference for exploratory pathway analysis. For network generation, default settings were used to identify molecules whose expression was significantly differentially regulated. These molecules were overlaid onto a global molecular network contained in the Ingenuity Knowledge Base. Networks of 'network-eligible molecules' were then algorithmically generated based on their connectivity. The functional analysis identified the biological functions and diseases that were most significant to the data set. A right-tailed Fisher's exact test was used to calculate p-values. Canonical pathway analysis identified the pathways from the IPA library that were most significant to the data set. Analysis was performed on all proteomics data, comparing healthy, mild OA, and severe OA groups and those proteins correlated to phospholipids.

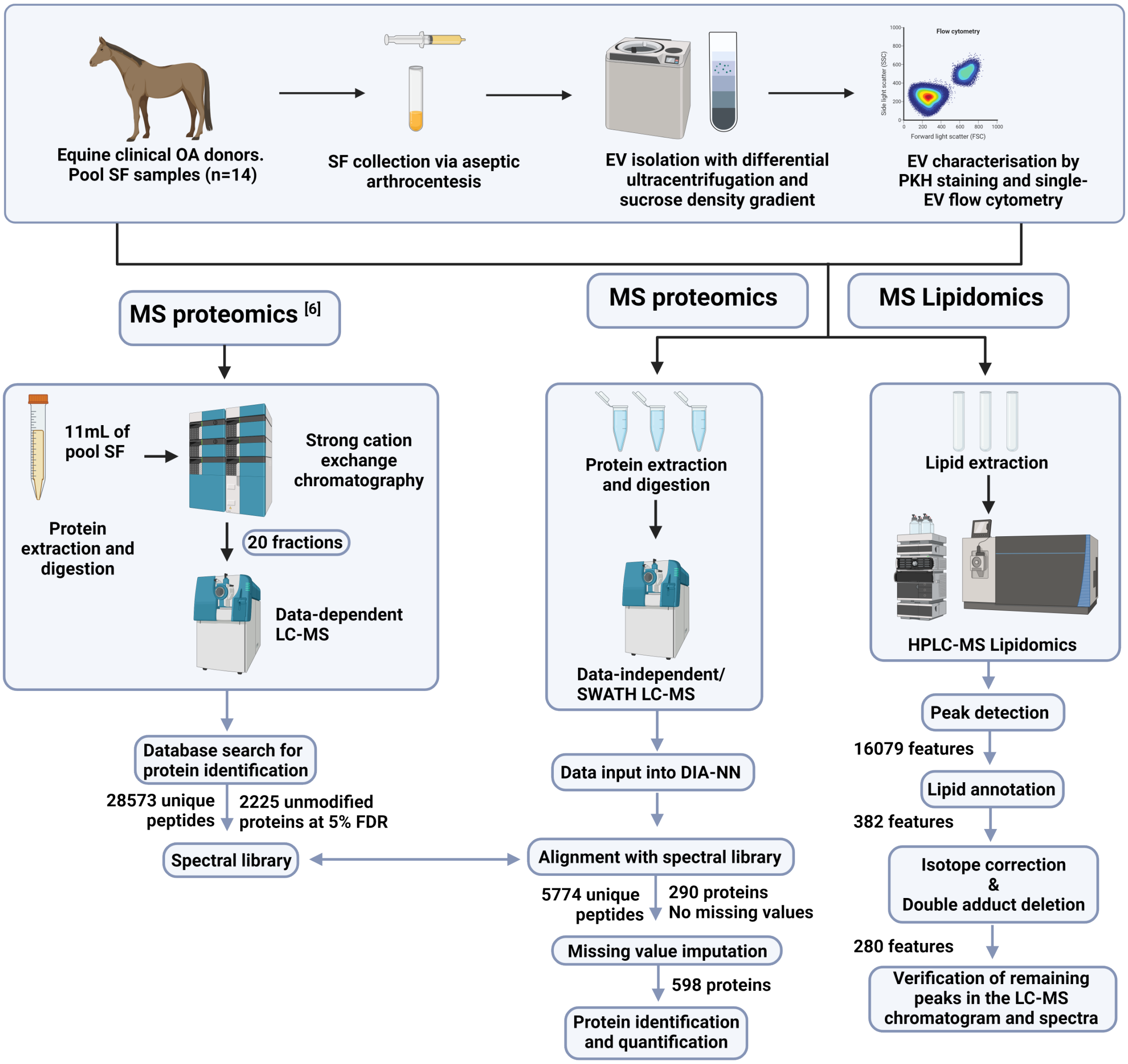

Suppl. Fig. 1 : Workflow for sample processing. OA was characterized following clinical and radiographic examinations. SF was collected by sterile arthrocentesis and spun to create cell-free SF. Forty-two donors were used to create 14 samples consisting of a pool of three unique biological samples with a volume of 5mL (Healthy n=7, mild OA n= 4, and severe OA n= 3). EVs were isolated from cell-free SF following differential ultracentrifugation with a sucrose density gradient [1]. EVs were stained using PKH and subsequently characterized following quantitative single EV-based high-resolution flow cytometry [4; 5]. The EV lipidome was probed following a chloroform and methanol lipid extraction [7], and mass spectrometry lipidomics was performed using a Fusion Orbitrap MS passing through a heated electrospray ionization [8]. The peaks were detected based on retention time, exact m/z-ratio, and, if present in at least 3 out of 13 samples (Healthy n=6, OA n=4, Advanced OA n=3). The features were annotated using an in-silico phospholipid database. The features were also selected to account for isotope distribution and adducts. The EV proteome was extracted using a urea lysis buffer, and proteins were subsequently digested on hydrophilic and hydrophobic magnetic carboxylate SpeedBeads, using trypsin/lyseC [12]. A data-independent proteomic approach was utilised in the form of Sequential Windowed Acquisition of all theoretical fragments (SWATH), and analysis was performed using a Triple TOF 6600. Statistical analysis was performed on respective datasets independently with Rstudio or Metaboanalys.

Table 2: Author Checklist: MIFlowCyt-Compliant Items.

Requirement	Requested Information
1.1. Purpose	To investigate extracellular vesicle biomarker discovery in synovial fluid from healthy, mild, and severe osteoarthritis (OA) equine samples by using a combined proteomics and lipidomics approach

1.2. Keywords	Osteoarthritis, synovial fluid, proteomics, lipidomics, equine, extracellular vesicles
1.3. Experiment variables	Healthy versus clinically relevant equine OA
1.4. Organisation name and address	Division Cell Biology, Metabolism & Cancer<br>Department of Biomolecular Health Sciences<br>Faculty of Veterinary Sciences<br>Utrecht University<br>Yalelaan 2, 3584 CM<br>Utrecht, The Netherlands
1.5. Primary contact name and email address	Prof. Dr. M.H.M. Wauben<br>M.H.M.Wauben@uu.nl
1.6. Date or time period of experiment	October 2022
1.7. Conclusions	The proteome and lipidome of SF-EVs are primarily positively correlated with activation of pathways related to chondrocyte dysregulation and inflammation. A series of phospholipids and proteins were proposed as alternatives for combined biomarker discovery, for example, PC O-32:3 and CD109.
1.8. Quality control measures	Procedural control for fluorescent stainings<br><br>Serial dilutions<br><br>Detergent treatment control
2.1.1.1. (2.1.2.1., 2.1.3.1.) Sample description	Synovial fluid from healthy equine joints, joints with diagnosed mild OA or severe OA
2.1.1.2. Biological sample source description	Synovial fluid
2.1.1.3. Biological sample source organism description	Horse (Equus caballus)
2.1.2.2. Environmental sample location	N/A
2.3. Sample treatment description	For detergent treatment, control samples were treated with 0.1% (v/v) triton X-100 (SERVA Electrophoresis GmbH, Heidelberg, Germany) final concentration for 30 seconds before reanalysis.
2.4. Fluorescence reagent(s) description	PKH67 (Sigma-Aldrich)
3.1. Instrument manufacturer	Becton Dickinson
3.2. Instrument model	BD Influx™ optimized instrument for detection of sub-micron-sized particles as described previously.
3.3. Instrument configuration and settings	BD Influx optimized to measure small particles. All configuration details can be found in previous publications [4; 5]. Briefly, samples were measured at a constant flow rate for 30 seconds using a fluorescence threshold on the 488 nm laser. The threshold level was set to detect 10-20 events per second when measuring a buffer control.
4.1. List-mode data files	All data files, including the quality control measure from 1.8, are available upon request.
4.2. Compensation description	No compensation was required due to instrument configuration.
4.3. Data transformation details	No data transformation was applied.
4.4.1. Gate description	No gates were applied
4.4.2. Gate statistics	The number of total events recorded in 30 seconds measurements are shown in the dot plots without any background correction.
4.4.3. Gate boundaries	N/A

Table 3: MIFlowCyt-EV framework.

1.1 Preanalytical variables conforming to MISEV guidelines	Yes, all relevant data has been submitted to EV-TRACK for transparent reporting and centralizing knowledge in extracellular vesicle research (EV-TRACK ID: EV230607).
1.2 Experimental design according to MIFlowCyt guidelines	Yes, MIFlowCyt checklist can be found as part of the supporting information of this manuscript in Supplementary Table 2.
2.1 Sample staining details	Yes, described in Materials and Methods.
2.2 Sample washing details	Yes, described in Materials and Methods.
2.3 Sample dilution details	Yes, described in Materials and Methods.
3.1 Buffer-only controls	Yes, relevant buffer controls were measured
3.2 Buffer with reagent controls	Yes. Data available upon request
3.3 Unstained controls	N/A
3.4 Isotype controls	N/A
3.5 Single-stained controls	N/A
3.6 Procedural controls	Yes. Available upon request
3.7 Serial dilutions	Yes, serial dilutions were performed in previous characterization experiments to determine the ideal dilution used in this study. Data available upon request
3.8 Detergent-treated controls	Yes, sensitivity to triton X-100 was determined in previous characterization experiments. Data available upon request
4.1 Trigger channel(s) and threshold(s)	The trigger channel used was on the fluorescent signal collected from the 488 nm laser (530/40 bandpass filter). The threshold level was set at 0.62, allowing an event rate of <20 events/second in a clean PBS sample. Additional details can be found in Materials and Methods.
4.2 Flow rate / volumetric quantification	Yes, low flow rate was kept constant and was measured for quantification purposes. The flow rate was estimated at 12.8 μL/min.
4.3 Fluorescence calibration	N/A
4.4 Scatter calibration	N/A
5.1 EV diameter/surface area/volume approximation	N/A
5.2 EV refractive index approximation	N/A
5.3 EV epitope number approximation	N/A
6.1 Completion of MIFlowCyt checklist	Yes, see Supplementary Table 2
6.2 Calibrated channel detection range	As shown in previous publications, equivalent to 100 FITC MESF. See description in Arkesteijn GJA et al.[5]
6.3 EV number/concentration	Yes, see Figure 1.
6.4 EV brightness	N/A
7.1 Sharing of data to a public repository	Yes, all experimental details about the biological sample preparation can be found in EV-TRACK. All data files are available upon request.

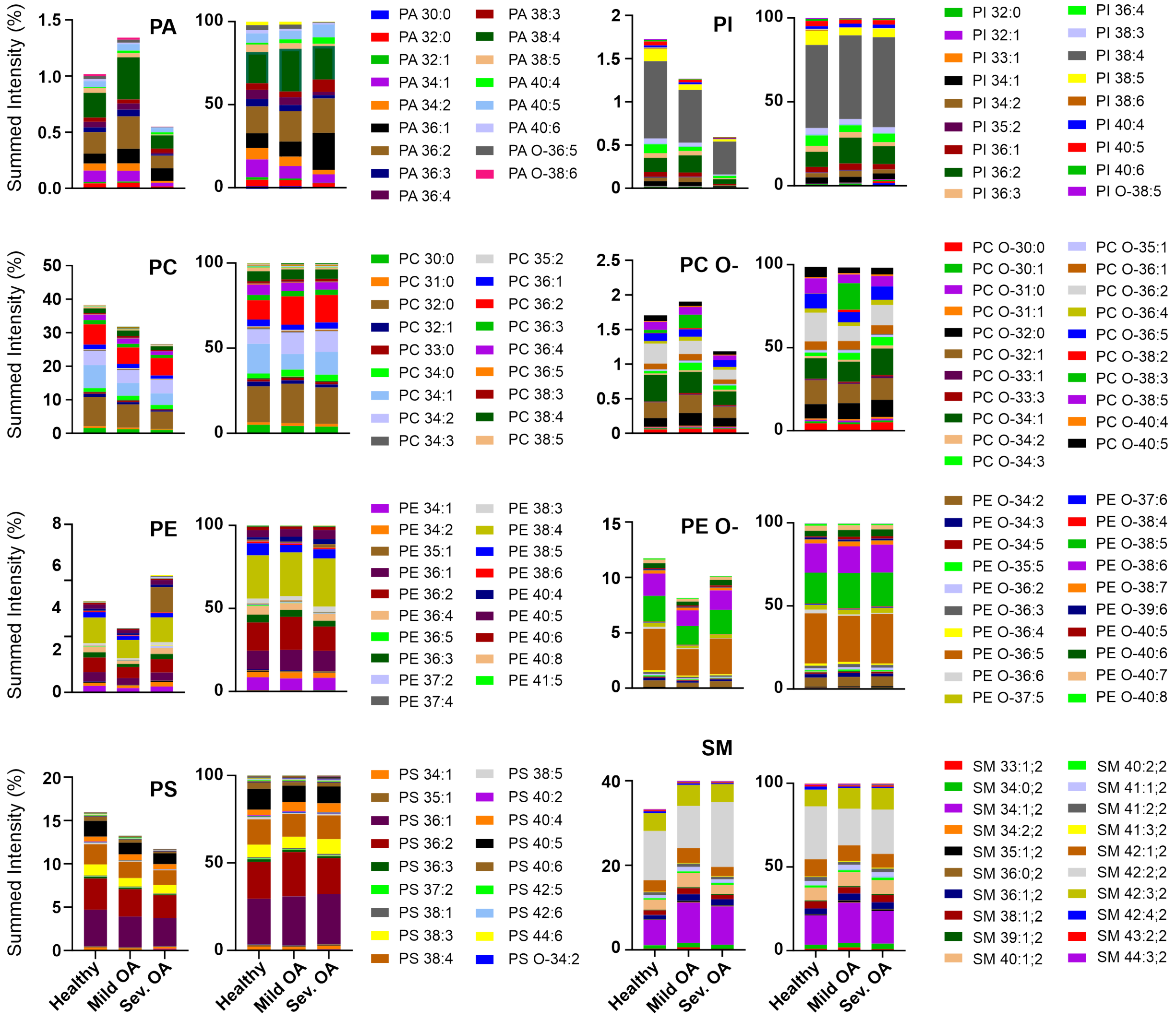

Suppl. Fig. 2 : Composition of individual lipid species in PA, PI, PC (O-), PE(O-), PS, and SM classes. Lipid composition of the most abundant lipid classes of SF-EVs from healthy, mild OA, or severe OA horse patients. The left stacked bars graph in each class shows the lipid species' amount in the overall lipidome. The immediately right stacked bars graph displays the normalized amount of lipid species in each class. Samples were normalised for each class and SF-EV group by expressing the lipid intensity as a fraction of the sum of lipid intensities. Lipids were obtained from 100,000g purified SF-EVs with sucrose density gradients. Healthy samples (n=6), mild OA (n=4), severe OA (n=3). Abbreviations: PA (phosphatidic acid), PC (ester-linked phosphatidylcholine), PC O- (ether-linked phosphatidylcholine), PE (ester-linked phosphatidylethanolamine), PE O- (ether-linked phosphatidylethanolamine), PI (phosphatidylinositol), PS (phosphatidylserine), SM (sphingomyelin).

Table 4: Significantly differentially expressed pathological functions, as identified by ingenuity pathway analysis for each comparison.

	Disease or Function Annotation	p-value
**Severe OA Vs mild OA**	Accumulation of macrophages	0.000414
	Stabilization of microtubules	0.000429
	Necrosis	0.000433
	Formation of microtubules	0.000563
	Translocation of microtubules	0.000582
	Outgrowth of microtubules	0.000582
	Quantity of filaments	0.00103
	Accumulation of microtubules	0.00146
	Conversion of neurons	0.00233
	Length of microtubules	0.00291
	Organization of microtubule organizing centers	0.0032
	Instability of microtubules	0.00349
	Regulation of actin cytoskeleton	0.00378
	Anchoring of microtubules	0.00436
	Activation of basophils	0.00552
	Injury of joint	0.00552
	Cell division of stem cells	0.00697
	Arrest in cell cycle progression of fibroblasts	0.00697
	Organization of basement membrane	0.00726
	Quantity of basophils	0.00755
	Induction of reactive oxygen species	0.00784
	Deposition of extracellular matrix	0.00784
	Quantity of microtubules	0.00842
	Abnormal morphology of basement membrane	0.00986
	Adhesion of macrophages	0.0107
	Activation of phagocytes	0.0119
	Complement activation	0.0171
	Phagocytosis of neutrophils	0.0185
	Organization of microtubules	0.0191
	Migration of cells	0.0198
	Polymerization of microtubules	0.0245
	Quantity of actin filaments	0.0259
	Adhesion of cell-associated matrix	0.0305
	Inflammation of organ	0.0315
	Apoptosis	0.0326
	Cell movement of phagocytes	0.0327
	Cell death of osteosarcoma cells	0.0389
**Severe OA vs healthy**	Organization of cytoskeleton	0.0000012
	Linkage of actin cytoskeleton	1.25E-06
	Decapping of actin filaments	4.15E-06
	Binding of actin cytoskeleton	8.71E-06
	Clathrin mediated endocytosis	0.0000248
	Phagocytosis	0.0000418
	Proliferation of immune cells	0.000126
	Endocytosis	0.000256
	Adhesion of phagocytes	0.000285
	Morphology of cytoskeleton	0.00032
	Remodeling of actin cytoskeleton	0.000369
	Rac protein signal transduction	0.000387
	Maturation of phagosomes	0.000583
	Formation of vesicles	0.000712
	Trafficking of exocytic vesicle	0.00133
	Association of phospholipid vesicles	0.00133

	Endocytosis of plasma membrane	0.00133
	Blebbing	0.00134
	Quantity of phagocytes	0.00151
	Quantity of reactive oxygen species	0.00168
	Formation of reactive oxygen species	0.00168
	Cell movement of connective tissue cells	0.00171
	Necrosis	0.0018
	Oxidative stress	0.0019
	Survival of synovial fibroblasts	0.002
	Initiation of autophagy of cells	0.002
	Proliferation of macrophages	0.00202
	Migration of phagocytes	0.00299
	Differentiation of phagocytes	0.00312
	Transmigration of phagocytes	0.00362
	Function of cytoskeleton	0.00399
	Accumulation of proteoglycan	0.00465
	Cell movement of neutrophils	0.00484
	Apoptosis of chondrocyte cell lines	0.00598
	Invasion of fibroblast-like synoviocytes	0.00598
	Organization of extracellular matrix	0.00598
	Migration of neutrophils	0.00625
	Invasion of extracellular matrix	0.00664
	Release of secretory vesicles	0.00664
	Migration of osteoclasts	0.00664
	Apoptosis	0.00669
	Fusion of osteoclasts	0.00862
	Fusion of osteoclast precursor cells	0.00928
	Movement of vesicles	0.00994
	Migration of fibroblast-like synoviocytes	0.0139
	Accumulation of extracellular matrix	0.0165
	Immune mediated inflammatory disease	0.0175
	Quantity of chondrocytes	0.0185
	Fibrosis	0.0189
	Cellular infiltration by macrophages	0.0194
	Morphology of bone	0.0195
	Systemic inflammation	0.0211
	Inflammation of organ	0.0231
	Development of chondrocytes	0.027
	Disruption of cytoskeleton	0.027
	Acute inflammation of tissue	0.0289
	Differentiation of connective tissue cells	0.031
	Osteoarthritis	0.0316
**Mild OA vs healthy**	Complement activation	0.000609
	Coalignment of microfilaments	0.000624
	Necrosis	0.000961
	Chondrogenesis of bone marrow stromal cells	0.00125
	Abnormal morphology of trabecular bone	0.00167
	Immune response of neutrophils	0.0017
	Development of articular cartilage	0.00374
	Morphogenesis of endochondral bone	0.00498
	Fusion of joint	0.00746
	Abnormal morphology of bone	0.00748
	Resorption of bone	0.00904
	Phagocytosis of phagocytes	0.00949
	Differentiation of osteoclasts	0.0116
	Injury of joint	0.0118

Morphology of bone	0.0163
Inflammation of joint	0.0169
Mineralization of extracellular matrix	0.0198
Activation of macrophages	0.0235
Cell movement of phagocytes	0.0236
Function of osteoblasts	0.0253
Osteoarthritis	0.028
Chronic inflammation	0.0338
Function of osteoclasts	0.038
Cell movement of macrophages	0.0381
Phagocytosis of neutrophils	0.0392
Cellular infiltration by phagocytes	0.0416

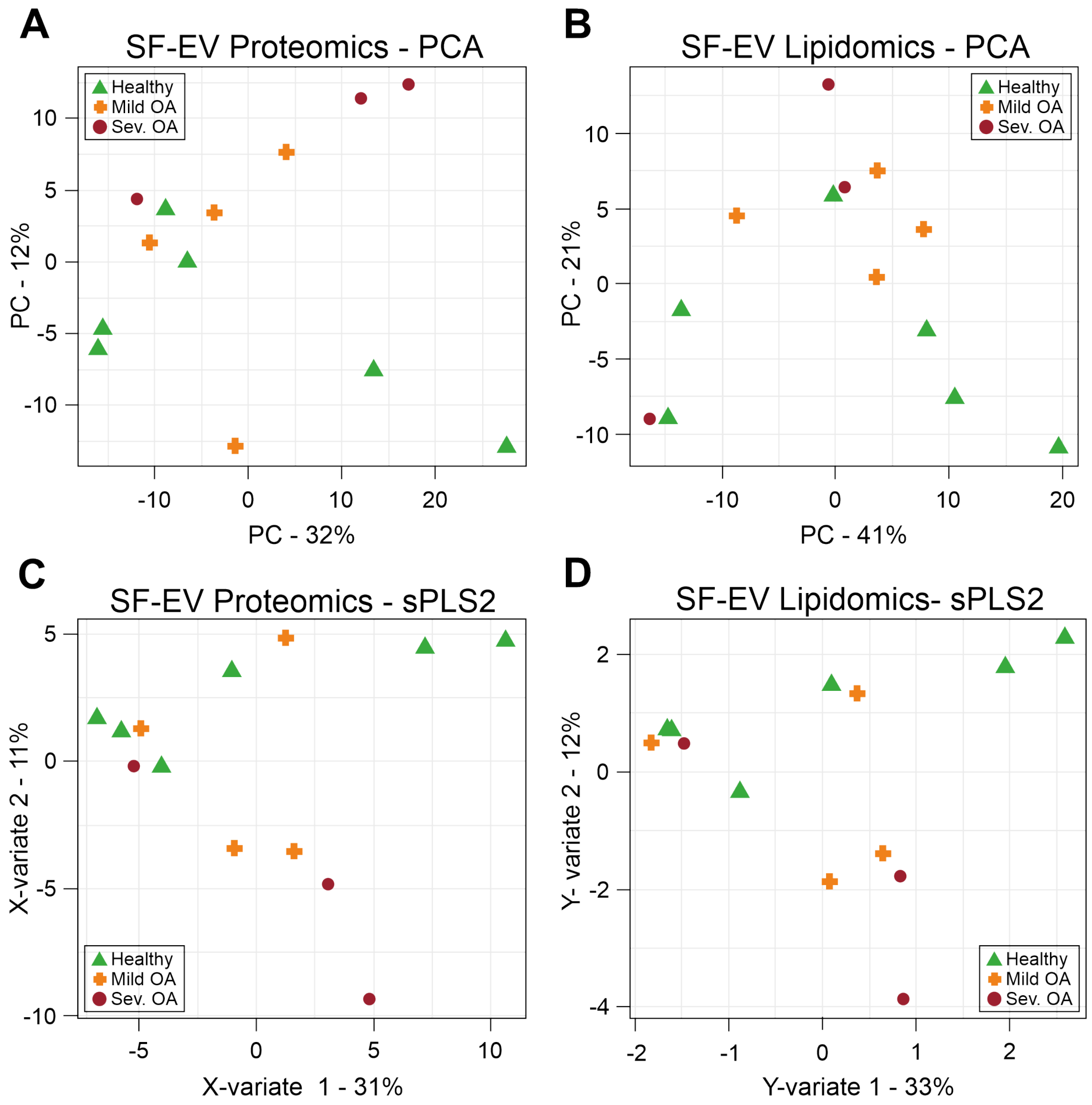

Suppl. Fig. 3 : Normalisation of proteomics and lipidomics data for integrated analysis. Healthy SF-EVs (green triangle, n=6), mild OA SF-EVs (orange cross, n=4), and severe OA SF-EVs (Sev. OA; red circle, n=3). Samples of both datasets were normalized by the sum. **A-B)** Principal component analysis (PCA) of the proteomics (A) and lipidomics (B) SF-EV datasets. **C-D)** Unsupervised multivariate Sparse Partial Least Squares regression (sPLS2) of the SF-EV samples in X-variate (for proteomics (C)) and Y-variate (for lipidomics (C)) components projected into the space spanned to the respective dataset.

Table 5: Candidate proteins for composite OA biomarker discovery.

	Protein	Membrane or cargo protein	Origin	Function
		**Group 1*– Downregulated proteins**		
**Enzymes (oxidative stress)**	Peroxiredoxin 1	Cargo [19]	Dendritic cells [20], spleen cells [21], osteoblasts [22], macrophages [23], thymus [24], pancreatic-β cells [25], oligodendrocytes [26],	Modulation of redox signalling events for cellular protection [27]
**Membrane proteins**	CD163 (Scavenger receptor cysteine-rich type 1 protein M130)	Transmembrane [28]	Macrophage [28; 29]	Receptor for clearance of oxidative and proinflammatory hemoglobin/haptoglobin complexes, stimulator of heme-oxygenase-1 and anti-inflammatory heme metabolites [28]
**Membrane traffic**	Caveolin 1	Cargo [30]	Ubiquitously expressed in all cell types [31]	Generation of caveolae (membrane curvatures) [30]
**Structural and cytoskeleton-related molecules**	Myosin regulatory light polypeptide 9	Cargo [32]	Smooth muscle, spleen, fibroblasts (all non-muscle tissues) [32], Astrocytes [33], hippocampus [34] cancer [35; 36]	Modulation of contractile activity of smooth muscle and non-muscle cells by phosphorylation [37]
	Tubulin alpha chain	Cargo [38]	Ubiquitously expressed in all cell types [38]	Cellular morphology formation and maintenance, and intracellular transport, chromosomal segregation [38]
**Chaperones**	Heat shock protein 90 alpha	Cargo [39]	Fibroblasts [40], pancreatic-β cells [25], oligodendrocytes [26], B cell [41], cancer [42–45]	Protein folding, maturation, and involvement in signal transduction and transcriptional regulation [46]
**Endocytosis, exocytosis, and trafficking**	Annexin A8	Cargo [47; 48]	Chondrocytes [48], Leukocytes [49], osteoclasts [50], lung, liver, kidney, skin, placenta, and cornea [51]	Controls sorting and transport of late endosomes [47], chondrocyte differentiation marker under normal endochondral ossification [48]
**Signal transduction**	Protein S100-A4	Cargo [52], secreted [53]	Chondrocytes [52], macrophages [53], fibroblasts, CD8+ T cells, monocytes, and eosinophils, cancer [54]	Regulation of calcium homeostasis, cell growth and motility, cell differentiation, and cell survival [52], promotion and induction of metastasis [54]
		**Group 2** – Upregulated proteins**		
**Metabolism**	Aldo-keto reductase family 7 member A2	Cargo	Cartilage [55], cardiomyocytes [56], brain, heart, kidney, liver, lung, prostate, skeletal muscle, small intestine, spleen [57]	Reduction of aldehydes and ketones to alcohols and metabolism of toxic aldehydes [56]

**Structural and cytoskeleton-related molecules**	Erythrocyte membrane protein band 4.1. like 2	Cargo [58]	B cells [59], endothelial cells[60], Dendritic cells [61], Mesenchymal stem cells [62], T cells [63]	Role in the attachment of cytoplasmic proteins to the membrane, part of the FERM complex (composed of the 4.1 protein, ezrin, radixin, and moesin) [58]
	Ezrin	Cargo [64]	Chondrocytes [65], fibroblast [66; 67], B cells [59], milk [68], Dendritic cells [61], oligodendrocytes [26], cancer cells [69; 70], macrophages [71], monocytes [72], T cells [73], synovial fluid [74] B cell [59; 75], milk [68],	Cross-linker between the actin cytoskeleton and the plasma membrane, part of the ERM complex (ezrin/radixin/moesin), also involved in signal transduction cell migration, and survival [64]
	Moesin	Cargo [76]	Dendritic cells [61], endothelial cells [77], neutrophils [78], synovial fluid [74]	Links cytoplasmic regions of integrins and modulate their function [79; 80]
	Fermitin family member 3	Cargo [79]	B cells [59], cancer cells [81; 82], Dendritic cells [61], endothelial cells [77], monocytes [72], neutrophils [78], red blood cells [83], platelets [84]	Bind to the cytoplasmic regions of integrins and modulate their function [79; 80]
	Actin alpha 2, smooth muscle ((α)-2 smooth muscle actin)	Cargo	Chondrocytes [85], monocytes and macrophages [86], fibroblasts [87]	Actin isoform contributes to cell-generated mechanical tension, cell structure, tissue remodelling and contraction [88]. Role in fibrosis [89–91]
	Vasodilator stimulated phosphoprotein	Cargo	B cells [59; 81], milk [68], dendritic cells [61], endothelial cells [77], monocytes [72], platelets [84; 92], T cells [73], cancer cells [82; 93; 94]	Associated with cell differentiation, mobility and tumour metastasis [95]
**Membrane proteins**	CD109	Transmembrane [96]	Cancer cells [82; 93; 94; 97], T cells [73], Dendritic cells [61], Mesenchymal stem cells [62], endothelial cells [60], synovial tissue [98]	Modulation of pathological processes, such as osteoporosis, fibrosis, and tumour metastasis [98], TGF-β co-receptor and signaling inhibitor of TGF-β in keratinocytes [99]
	Thy-1 antigen/CD90	Transmembrane [100]	T cells, NK cells, innate lymphoid cells [101], fibroblasts [102], Mesenchymal stem cells [62], cancer cells [82], endothelial cells, epithelial cells, neurons [100]	Involved in cancer development and metastasis, cell proliferation, differentiation, cell migration, apoptosis, mechanotransduction and cell adhesion[100; 103]
**Endocytosis, exocytosis and trafficking**	RAB8B, member RAS oncogene family	Cargo and secreted	Sperm[104], glioma cells [105], and SARS-CoV-2 sera [106]	Involved in membrane trafficking and the establishment of the Golgi apparatus [104]

**Signal transduction**	Rac family small GTPase 1	Cargo and secreted	Hepatic cells [107], range of eukaryotic cells [108], equine synovial fluid and plasma [109]	Activated GTPases are involved in cellular proliferation, differentiation, motility, survival, and apoptosis [108]
**Transmembrane transport**	Potassium channel tetramerisation domain containing 12	Membrane	Cancer cells[110; 111], Serum [112]	Involved in neuronal excitability through GABBA receptor signalling. Also found to suppress Wnt/Notch signaling, stem cell factors, and chromatin remodelers [113]. Associated with tetramerization and gating of ion channels, cytoskeleton regulation, and transcriptional repression [114]

* Proteins from group 1 correlated to the following phospholipids: PC 34:4, PI 38:6, PI 32:1.
** Proteins from group 2 correlated to the following phospholipids: PC O-32:3, SM 36:0;2, SM 41:3,2.

References

[1] J. Boere, C. H. A. van de Lest, S. F. W. M. Libregts, G. J. A. Arkesteijn, W. J. C. Geerts, E. N. M. Nolte-'t Hoen, J. Malda, P. R. van Weeren, and M. H. M. Wauben, "Synovial fluid pretreatment with hyaluronidase facilitates isolation of CD44+ extracellular vesicles," *Journal of Extracellular Vesicles*, vol. 5, p. 31751, Jan. 2016.

[2] L. Varela and C. H. van de Lest, "Acute joint inflammation induces a sharp increase in the number of synovial fluid EVs and modifies their phospholipid profile," May 2023.

[3] J. Van Deun, P. Mestdagh, P. Agostinis, Akay, S. Anand, J. Anckaert, Z. A. Martinez, T. Baetens, E. Beghein, L. Bertier, G. Berx, J. Boere, S. Boukouris, M. Bremer, D. Buschmann, J. B. Byrd, C. Casert, L. Cheng, A. Cmoch, D. Daveloose, E. De Smedt, S. Demirsoy, V. Depoorter, B. Dhondt, T. A. P. Driedonks, A. Dudek, A. Elsharawy, I. Floris, A. D. Foers, K. Gärtner, A. D. Garg, E. Geeurickx, J. Gettemans, F. Ghazavi, B. Giebel, T. G. Kormelink, G. Hancock, H. Helsmoortel, A. F. Hill, V. Hyenne, H. Kalra, D. Kim, J. Kowal, S. Kraemer, P. Leidinger, C. Leonelli, Y. Liang, L. Lippens, S. Liu, A. Lo Cicero, S. Martin, S. Mathivanan, P. Mathiyalagan, T. Matusek, G. Milani, M. Monguió-Tortajada, L. M. Mus, D. C. Muth, A. Németh, E. N. M. Nolte-'t Hoen, L. O'Driscoll, R. Palmulli, M. W. Pfaffl, B. Primdal-Bengtson, E. Romano, Q. Rousseau, S. Sahoo, N. Sampaio, M. Samuel, B. Scicluna, B. Soen, A. Steels, J. V. Swinnen, M. Takatalo, S. Thaminy, C. Théry, J. Tulkens, I. Van Audenhove, S. van der Grein, A. Van Goethem, M. J. van Herwijnen, G. Van Niel, N. Van Roy, A. R. Van Vliet, N. Vandamme, S. Vanhauwaert, G. Vergauwen, F. Verweij, A. Wallaert, M. Wauben, K. W. Witwer, M. I. Zonneveld, O. De Wever, J. Vandesompele, and A. Hendrix, "EV-TRACK: transparent reporting and centralizing knowledge in extracellular vesicle research," *Nat Methods*, vol. 14, pp. 228–232, Mar. 2017. Number: 3 Publisher: Nature Publishing Group.

[4] E. J. van der Vlist, E. N. M. Nolte-'t Hoen, W. Stoorvogel, G. J. A. Arkesteijn, and M. H. M. Wauben, "Fluorescent labeling of nano-sized vesicles released by cells and subsequent quantitative and qualitative analysis by high-resolution flow cytometry," *Nat Protoc*, vol. 7, pp. 1311–1326, July 2012.

[5] G. J. A. Arkesteijn, E. Lozano-Andrés, S. F. W. M. Libregts, and M. H. M. Wauben, "Improved Flow Cytometric Light Scatter Detection of Submicron-Sized Particles by Reduction of Optical Background Signals," *Cytometry Part A*, vol. 97, no. 6, pp. 610–619, 2020.

[6] J. A. Welsh, E. Van Der Pol, G. J. Arkesteijn, M. Bremer, A. Brisson, F. Coumans, F. Dignat-George, E. Duggan, I. Ghiran, B. Giebel, A. Görgens, A. Hendrix, R. Lacroix, J. Lannigan, S. F. Libregts, E. Lozano-Andrés, A. Morales-Kastresana, S. Robert, L. De Rond, T. Tertel, J. Tigges, O. De Wever, X. Yan, R. Nieuwland, M. H. Wauben, J. P. Nolan, and J. C. Jones, "MIFlowCyt-EV: a framework for standardized reporting of extracellular vesicle flow cytometry experiments," *Journal of Extracellular Vesicles*, vol. 9, p. 1713526, Sept. 2020.

[7] E. G. Bligh and W. J. Dyer, "A rapid method of total lipid extraction and purification," *Can. J. Biochem. Physiol.*, vol. 37, pp. 911–917, Aug. 1959.

[8] A. Jeucken, M. R. Molenaar, C. H. van de Lest, J. W. Jansen, J. B. Helms, and J. F. Brouwers, "A Comprehensive Functional Characterization of Escherichia coli Lipid Genes," *Cell Reports*, vol. 27, pp. 1597–1606.e2, Apr. 2019.

[9] M. C. Chambers, B. Maclean, R. Burke, D. Amodei, D. L. Ruderman, S. Neumann, L. Gatto, B. Fischer, B. Pratt, J. Egertson, K. Hoff, D. Kessner, N. Tasman, N. Shulman, B. Frewen, T. A. Baker, M.-Y. Brusniak, C. Paulse, D. Creasy, L. Flashner, K. Kani, C. Moulding, S. L. Seymour, L. M. Nuwaysir, B. Lefebvre, F. Kuhlmann, J. Roark, P. Rainer, S. Detlev, T. Hemenway, A. Huhmer, J. Langridge, B. Connolly, T. Chadick, K. Holly, J. Eckels, E. W. Deutsch, R. L. Moritz, J. E. Katz, D. B. Agus, M. MacCoss, D. L. Tabb, and P. Mallick, "A cross-platform toolkit for mass spectrometry and proteomics," *Nat Biotechnol*, vol. 30, pp. 918–920, Oct. 2012.

[10] H. P. Benton, E. J. Want, and T. M. D. Ebbels, "Correction of mass calibration gaps in liquid chromatography–mass spectrometry metabolomics data," *Bioinformatics*, vol. 26, pp. 2488–2489, Oct. 2010.

[11] L. Varela, E. Clarke, and C. H. van de Lest, "Proteome and phospholipidome interrelationship of synovial fluid-derived extracellular vesicles in equine osteoarthritis: An exploratory 'multi-omics' study towards combined biomarkers," July 2023.

[12] E. J. Clarke, J. R. Anderson, and M. J. Peffers, "Nuclear magnetic resonance spectroscopy of biofluids for osteoarthritis," *British Medical Bulletin*, vol. 137, pp. 28–41, Mar. 2021.

[13] E. J. Clarke, E. Johnson, E. Caamaño Gutierrez, C. Andersen, L. C. Berg, R. E. Jenkins, C. Lindegaard, K. Uvebrant, E. Lundgren-Åkerlund, A. Turlo, V. James, S. Jacobsen, and M. J. Peffers, "Temporal extracellular vesicle protein changes following intraarticular treatment with integrin 101-selected mesenchymal stem cells in equine osteoarthritis," *Frontiers in Veterinary Science*, vol. 9, 2022.

[14] M. Pathan, P. Fonseka, S. V. Chitti, T. Kang, R. Sanwlani, J. Van Deun, A. Hendrix, and S. Mathivanan, "Vesiclepedia 2019: a compendium of RNA, proteins, lipids and metabolites in extracellular vesicles," *Nucleic Acids Research*, vol. 47, pp. D516–D519, Jan. 2019.

[15] Z. Pang, J. Chong, G. Zhou, D. A. de Lima Morais, L. Chang, M. Barrette, C. Gauthier, P.- Jacques, S. Li, and J. Xia, "MetaboAnalyst 5.0: narrowing the gap between raw spectra and functional insights," *Nucleic Acids Research*, vol. 49, pp. W388–W396, July 2021.

[16] R. Team, "RStudio: Integrated Development Environment for R," 2020.

[17] Z. Gu, R. Eils, and M. Schlesner, "Complex heatmaps reveal patterns and correlations in multidimensional genomic data," *Bioinformatics*, vol. 32, pp. 2847–2849, Sept. 2016.

[18] F. Rohart, B. Gautier, A. Singh, and K.-A. Lê Cao, "mixOmics: An R package for 'omics feature selection and multiple data integration," *PLoS Comput Biol*, vol. 13, p. e1005752, Nov. 2017.

[19] C. A. Neumann, J. Cao, and Y. Manevich, "Peroxiredoxin 1 and its role in cell signaling," *Cell Cycle*, vol. 8, pp. 4072–4078, Dec. 2009.

[20] C. Théry, M. Boussac, P. Véron, P. Ricciardi-Castagnoli, G. Raposo, J. Garin, and S. Amigorena, "Proteomic Analysis of Dendritic Cell-Derived Exosomes: A Secreted Subcellular Compartment Distinct from Apoptotic Vesicles1," *The Journal of Immunology*, vol. 166, pp. 7309–7318, June 2001.

[21] M. Zöller, K. Zhao, N. N. Kutlu, N. Bauer, J. Provaznik, T. Hackert, and M. Schnölzer, "Immunoregulatory Effects of Myeloid-Derived Suppressor Cell Exosomes in Mouse Model of Autoimmune Alopecia Areata," *Front Immunol*, vol. 9, p. 1279, June 2018.

[22] Z. Xiao, C. E. Camalier, K. Nagashima, K. C. Chan, D. A. Lucas, M. J. d. l. Cruz, M. Gignac, S. Lockett, H. J. Issaq, T. D. Veenstra, T. P. Conrads, and G. R. Beck Jr., "Analysis of the extracellular matrix vesicle proteome in mineralizing osteoblasts," *Journal of Cellular Physiology*, vol. 210, no. 2, pp. 325–335, 2007.

[23] K. Hassani and M. Olivier, "Immunomodulatory Impact of Leishmania-Induced Macrophage Exosomes: A Comparative Proteomic and Functional Analysis," *PLoS Negl Trop Dis*, vol. 7, p. e2185, May 2013.

[24] L. Turiák, P. Misják, T. G. Szabó, B. Aradi, K. Pálóczi, O. Ozohanics, L. Drahos, Kittel, A. Falus, E. I. Buzás, and K. Vékey, "Proteomic characterization of thymocyte-derived microvesicles and apoptotic bodies in BALB/c mice," *Journal of Proteomics*, vol. 74, pp. 2025–2033, Sept. 2011.

[25] H. S. Lee, J. Jeong, and K.-J. Lee, "Characterization of vesicles secreted from insulinoma NIT-1 cells," *J Proteome Res*, vol. 8, pp. 2851–2862, June 2009.

[26] E.-M. Krämer-Albers, N. Bretz, S. Tenzer, C. Winterstein, W. Möbius, H. Berger, K.-A. Nave, H. Schild, and J. Trotter, "Oligodendrocytes secrete exosomes containing major myelin and stress-protective proteins: Trophic support for axons?," *PROTEOMICS – Clinical Applications*, vol. 1, no. 11, pp. 1446–1461, 2007.

[27] J. A. Collins, S. T. Wood, K. J. Nelson, M. A. Rowe, C. S. Carlson, S. Chubinskaya, L. B. Poole, C. M. Furdui, and R. F. Loeser, "Oxidative Stress Promotes Peroxiredoxin Hyperoxidation and Attenuates Pro-survival Signaling in Aging Chondrocytes*," *Journal of Biological Chemistry*, vol. 291, pp. 6641–6654, Mar. 2016.

[28] A. Etzerodt and S. K. Moestrup, "CD163 and Inflammation: Biological, Diagnostic, and Therapeutic Aspects," *Antioxid Redox Signal*, vol. 18, pp. 2352–2363, June 2013.

[29] M. Qu, H. Zhu, and X. Zhang, "Extracellular vesicle-mediated regulation of macrophage polarization in bacterial infections," *Frontiers in Microbiology*, vol. 13, 2022.

[30] L. Simón, A. Campos, L. Leyton, and A. F. G. Quest, "Caveolin-1 function at the plasma membrane and in intracellular compartments in cancer," *Cancer Metastasis Rev*, vol. 39, no. 2, pp. 435–453, 2020.

[31] Z. C. Nwosu, M. P. Ebert, S. Dooley, and C. Meyer, "Caveolin-1 in the regulation of cell metabolism: a cancer perspective," *Molecular Cancer*, vol. 15, p. 71, Nov. 2016.

[32] I. Park, C. Han, S. Jin, B. Lee, H. Choi, J. Kwon, D. Kim, J. Kim, E. Lifirsu, W. Park, Z. Park, D. Kim, and C. Cho, "Myosin regulatory light chains are required to maintain the stability of myosin II and cellular integrity," *Biochemical Journal*, vol. 434, pp. 171–180, Feb. 2011.

[33] S. Hallal, D. M. Mallawaaratchy, H. Wei, S. Ebrahimkhani, B. W. Stringer, B. W. Day, A. W. Boyd, G. J. Guillemin, M. E. Buckland, and K. L. Kaufman, "Extracellular Vesicles Released by Glioblastoma Cells Stimulate Normal Astrocytes to Acquire a Tumor-Supportive Phenotype Via p53 and MYC Signaling Pathways," *Mol Neurobiol*, vol. 56, pp. 4566–4581, June 2019.

[34] B. Xu, Y. Gao, S. Zhan, F. Xiong, W. Qiu, X. Qian, T. Wang, N. Wang, D. Zhang, Q. Yang, R. Wang, X. Bao, W. Dou, R. Tian, S. Meng, W.-P. Gai, Y. Huang, X.-X. Yan, W. Ge, and C. Ma, "Quantitative protein profiling of hippocampus during human aging," *Neurobiology of Aging*, vol. 39, pp. 46–56, Mar. 2016.

[35] M. Mujammami, M. Rafiullah, A. A. Alfadda, K. Akkour, I. O. Alanazi, A. Masood, M. Musambil, H. Alhalal, M. Arafah, A. M. A. Rahman, and H. Benabdelkamel, "Proteomic Analysis of Endometrial Cancer Tissues from Patients with Type 2 Diabetes Mellitus," *Life (Basel)*, vol. 12, p. 491, Mar. 2022.

[36] K. Matsushita, S. Kobayashi, H. Akita, M. Konno, A. Asai, T. Noda, Y. Iwagami, T. Asaoka, K. Gotoh, M. Mori, Y. Doki, H. Eguchi, and H. Ishii, "Clinicopathological significance of MYL9 expression in pancreatic ductal adenocarcinoma," *Cancer Rep (Hoboken)*, vol. 5, p. e1582, Nov. 2021.

[37] C. C. Kumar, S. R. Mohan, P. J. Zavodny, S. K. Narula, and P. J. Leibowitz, "Characterization and differential expression of human vascular smooth muscle myosin light chain 2 isoform in nonmuscle cells," *Biochemistry*, vol. 28, pp. 4027–4035, May 1989.

[38] I. Gasic, "Regulation of Tubulin Gene Expression: From Isotype Identity to Functional Specialization," *Frontiers in Cell and Developmental Biology*, vol. 10, 2022.

[39] N. C. Dempsey, F. Leoni, H. E. Ireland, C. Hoyle, and J. H. H. Williams, "Differential heat shock protein localization in chronic lymphocytic leukemia," *J Leukoc Biol*, vol. 87, pp. 467–476, Mar. 2010.

[40] V. Luga, L. Zhang, A. M. Viloria-Petit, A. A. Ogunjimi, M. R. Inanlou, E. Chiu, M. Buchanan, A. N. Hosein, M. Basik, and J. L. Wrana, "Exosomes Mediate Stromal Mobilization of Autocrine Wnt-PCP Signaling in Breast Cancer Cell Migration," *Cell*, vol. 151, pp. 1542–1556, Dec. 2012.

[41] A. Clayton, A. Turkes, H. Navabi, M. D. Mason, and Z. Tabi, "Induction of heat shock proteins in B-cell exosomes," *J Cell Sci*, vol. 118, pp. 3631–3638, Aug. 2005.

[42] W. Wei, J. Zhou, L. Chen, H. Liu, F. Zhang, J. Li, S. Ning, S. Li, C. Wang, Y. Huang, C. Zou, and L. Zhang, "Plasma Levels of Heat Shock Protein 90 Alpha Associated With Colorectal Cancer Development," *Front Mol Biosci*, vol. 8, p. 684836, 2021.

[43] B. Zhong, J. Shen, C. Zhang, G. Zhou, Y. Yu, E. Qin, J. Tang, D. Wu, and X. Liang, "Plasma Heat Shock Protein 90 Alpha: A Valuable Predictor of Early Chemotherapy Effectiveness in Advanced Non-Small-Cell Lung Cancer," *Med Sci Monit*, vol. 27, p. e924778, Jan. 2021.

[44] E. Bria, J. Furlanetto, L. Carbognin, M. Brunelli, C. Caliolo, R. Nortilli, F. Massari, S. Pedron, E. Manfrin, F. Pellini, F. Bonetti, I. Sperduti, G. P. Pollini, A. Scarpa, and G. Tortora, "Human epidermal growth factor receptor 2-positive breast cancer: heat shock protein 90 overexpression, Ki67 proliferative index, and topoisomerase II-co-amplification as predictors of pathologic complete response to neoadjuvant chemotherapy with trastuzumab and docetaxel," *Clin Breast Cancer*, vol. 15, pp. 16–23, Feb. 2015.

[45] S. M. Mousavi, S. Hosseindoost, S. M. A. Mahdian, N. Vousooghi, A. Rajabi, A. Jafari, A. Ostadian, M. R. Hamblin, M. Hadjighassem, and H. Mirzaei, "Exosomes released from U87 glioma cells treated with curcumin and/or temozolomide produce apoptosis in naive U87 cells," *Pathology - Research and Practice*, vol. 245, p. 154427, May 2023.

[46] V. Calamia, M. C. de Andrés, N. Oreiro, C. Ruiz-Romero, and F. J. Blanco, "Hsp90 inhibition modulates nitric oxide production and nitric oxide-induced apoptosis in human chondrocytes," *BMC Musculoskelet Disord*, vol. 12, p. 237, Oct. 2011.

[47] V. Goebeler, M. Poeter, D. Zeuschner, V. Gerke, and U. Rescher, "Annexin A8 Regulates Late Endosome Organization and Function," *Mol Biol Cell*, vol. 19, pp. 5267–5278, Dec. 2008.

[48] A. H. White, R. E. B. Watson, B. Newman, A. J. Freemont, and G. A. Wallis, "Annexin VIII Is Differentially Expressed by Chondrocytes in the Mammalian Growth Plate During Endochondral Ossification and in Osteoarthritic Cartilage," *Journal of Bone and Mineral Research*, vol. 17, no. 10, pp. 1851–1858, 2002.

[49] M. Poeter, I. Brandherm, J. Rossaint, G. Rosso, V. Shahin, B. V. Skryabin, A. Zarbock, V. Gerke, and U. Rescher, "Annexin A8 controls leukocyte recruitment to activated endothelial cells via cell surface delivery of CD63," *Nat Commun*, vol. 5, p. 3738, Apr. 2014. Number: 1 Publisher: Nature Publishing Group.

[50] T. N. Crotti, R. P. O'Sullivan, Z. Shen, M. R. Flannery, R. J. Fajardo, F. P. Ross, S. R. Goldring, and K. P. McHugh, "Bone matrix regulates osteoclast differentiation and annexin A8 gene expression," *Journal of Cellular Physiology*, vol. 226, no. 12, pp. 3413–3421, 2011.

[51] T. Grewal, C. Rentero, C. Enrich, M. Wahba, C. A. Raabe, and U. Rescher, "Annexin Animal Models—From Fundamental Principles to Translational Research," *International Journal of Molecular Sciences*, vol. 22, p. 3439, Jan. 2021. Number: 7 Publisher: Multidisciplinary Digital Publishing Institute.

[52] R. R. Yammani, "S100 proteins in cartilage: Role in arthritis," *Biochim Biophys Acta*, vol. 1822, pp. 600–606, Apr. 2012.

[53] L. Chen, J. Li, J. Zhang, C. Dai, X. Liu, J. Wang, Z. Gao, H. Guo, R. Wang, S. Lu, F. Wang, H. Zhang, H. Chen, X. Fan, S. Wang, and Z. Qin, "S100A4 promotes liver fibrosis via activation of hepatic stellate cells," *J Hepatol*, vol. 62, pp. 156–164, Jan. 2015.

[54] N. Ambartsumian, J. Klingelhöfer, and M. Grigorian, "The Multifaceted S100A4 Protein in Cancer and Inflammation," in *Calcium-Binding Proteins of the EF-Hand Superfamily: From Basics to Medical Applications* (C. W. Heizmann, ed.), Methods in Molecular Biology, pp. 339–365, New York, NY: Springer, 2019.

[55] J.-B. Vincourt, F. Lionneton, G. Kratassiouk, F. Guillemin, P. Netter, D. Mainard, and J. Magdalou, "Establishment of a Reliable Method for Direct Proteome Characterization of Human Articular Cartilage*," *Molecular & Cellular Proteomics*, vol. 5, pp. 1984–1995, Oct. 2006.

[56] A. Quiñones-Lombraña, A. Intini, and J. G. Blanco, "Insights into the transcriptional regulation of the anthracycline reductase AKR7A2 in human cardiomyocytes," *Toxicol Lett*, vol. 307, pp. 11–16, June 2019.

[57] T. O'connor, L. S. Ireland, D. J. Harrison, and J. D. Hayes, "Major differences exist in the function and tissue-specific expression of human aflatoxin B1 aldehyde reductase and the principal human aldo-keto reductase AKR1 family members," *Biochem J*, vol. 343 Pt 2, pp. 487–504, Oct. 1999.

[58] A. H. Chishti, A. C. Kim, S. M. Marfatia, M. Lutchman, M. Hanspal, H. Jindal, S.-C. Liu, P. S. Low, G. A. Rouleau, N. Mohandas, J. A. Chasis, J. G. Conboy, P. Gascard, Y. Takakuwa, S.-C. Huang, E. J. Benz Jr, A. Bretscher, R. G. Fehon, J. F. Gusella, V. Ramesh, F. Solomon, V. T. Marchesi, S. Tsukita, S. Tsukita, M. Arpin, D. Louvard, N. K. Tonks, J. M. Anderson, A. S. Fanning, P. J. Bryant, D. F. Woods, and K. B. Hoover, "The FERM domain: a unique module involved in the linkage of cytoplasmic proteins to the membrane," *Trends in Biochemical Sciences*, vol. 23, pp. 281–282, Aug. 1998.

[59] S. I. Buschow, B. W. M. van Balkom, M. Aalberts, A. J. R. Heck, M. Wauben, and W. Stoorvogel, "MHC class II-associated proteins in B-cell exosomes and potential functional implications for exosome biogenesis," *Immunology & Cell Biology*, vol. 88, no. 8, pp. 851–856, 2010.

[60] O. G. de Jong, M. C. Verhaar, Y. Chen, P. Vader, H. Gremmels, G. Posthuma, R. M. Schiffelers, M. Gucek, and B. W. M. van Balkom, "Cellular stress conditions are reflected in the protein and RNA content of endothelial cell-derived exosomes," *J Extracell Vesicles*, vol. 1, 2012.

[61] J. Kowal, G. Arras, M. Colombo, M. Jouve, J. P. Morath, B. Primdal-Bengtson, F. Dingli, D. Loew, M. Tkach, and C. Théry, "Proteomic comparison defines novel markers to characterize heterogeneous populations of extracellular vesicle subtypes," *Proc Natl Acad Sci U S A*, vol. 113, pp. E968–977, Feb. 2016.

[62] H.-S. Kim, D.-Y. Choi, S. J. Yun, S.-M. Choi, J. W. Kang, J. W. Jung, D. Hwang, K. P. Kim, and D.-W. Kim, "Proteomic Analysis of Microvesicles Derived from Human Mesenchymal Stem Cells," *J. Proteome Res.*, vol. 11, pp. 839–849, Feb. 2012. Publisher: American Chemical Society.

[63] A. Németh, N. Orgovan, B. W. Sódar, X. Osteikoetxea, K. Pálóczi, K. Szabó-Taylor, K. V. Vukman, Kittel, L. Turiák, Z. Wiener, S. Tóth, L. Drahos, K. Vékey, R. Horvath, and E. I. Buzás, "Antibiotic-induced release of small extracellular vesicles (exosomes) with surface-associated DNA," *Sci Rep*, vol. 7, p. 8202, Aug. 2017.

[64] K. Kawaguchi and S. Asano, "Pathophysiological Roles of Actin-Binding Scaffold Protein, Ezrin," *Int J Mol Sci*, vol. 23, p. 3246, Mar. 2022.

[65] B. Housmans, M. Neefjes, D. Surtel, M. Vitík, A. Cremers, L. Van Rhijn, P. Van Der Kraan, G. Van Den Akker, and T. Welting, "Synovial fluid from end-stage osteoarthritis induces proliferation and fibrosis of articular chondrocytes via MAPK and RhoGTPase signaling," *Osteoarthritis and Cartilage*, vol. 30, pp. 862–874, June 2022.

[66] C. T. Quang, A. Gautreau, M. Arpin, and R. Treisman, "Ezrin function is required for ROCK-mediated fibroblast transformation by the Net and Dbl oncogenes," *The EMBO Journal*, vol. 19, pp. 4565–4576, Sept. 2000. Publisher: John Wiley & Sons, Ltd.

[67] C. Quan, Y. Yan, Z. Qin, Z. Lin, and T. Quan, "Ezrin regulates skin fibroblast size/mechanical properties and YAP-dependent proliferation," *J Cell Commun Signal*, vol. 12, pp. 549–560, Sept. 2018.

[68] M. J. C. van Herwijnen, M. I. Zonneveld, S. Goerdayal, E. N. M. Nolte-'t Hoen, J. Garssen, B. Stahl, A. F. Maarten Altelaar, F. A. Redegeld, and M. H. M. Wauben, "Comprehensive Proteomic Analysis of Human Milk-derived Extracellular Vesicles Unveils a Novel Functional Proteome Distinct from Other Milk Components," *Mol Cell Proteomics*, vol. 15, pp. 3412–3423, Nov. 2016.

[69] D.-S. Choi, J.-M. Lee, G. W. Park, H.-W. Lim, J. Y. Bang, Y.-K. Kim, K.-H. Kwon, H. J. Kwon, K. P. Kim, and Y. S. Gho, "Proteomic analysis of microvesicles derived from human colorectal cancer cells," *J Proteome Res*, vol. 6, pp. 4646–4655, Dec. 2007.

[70] J. Paggetti, F. Haderk, M. Seiffert, B. Janji, U. Distler, W. Ammerlaan, Y. J. Kim, J. Adam, P. Lichter, E. Solary, G. Berchem, and E. Moussay, "Exosomes released by chronic lymphocytic leukemia cells induce the transition of stromal cells into cancer-associated fibroblasts," *Blood*, vol. 126, pp. 1106–1117, Aug. 2015.

[71] I. Kadiu, P. Narayanasamy, P. K. Dash, W. Zhang, and H. E. Gendelman, "Biochemical and Biologic Characterization of Exosomes and Microvesicles as Facilitators of HIV-1 Infection in Macrophages," *J Immunol*, vol. 189, pp. 744–754, July 2012.

[72] M. Bernimoulin, E. K. Waters, M. Foy, B. M. Steele, M. Sullivan, H. Falet, M. T. Walsh, N. Barteneva, J.-G. Geng, J. H. Hartwig, P. B. Maguire, and D. D. Wagner, "Differential stimulation of monocytic cells results in distinct populations of microparticles," *J Thromb Haemost*, vol. 7, pp. 1019–1028, June 2009.

[73] D. Perez-Hernandez, C. Gutiérrez-Vázquez, I. Jorge, S. López-Martín, A. Ursa, F. Sánchez-Madrid, J. Vázquez, and M. Yáñez-Mó, "The intracellular interactome of tetraspanin-enriched microdomains reveals their function as sorting machineries toward exosomes," *J Biol Chem*, vol. 288, pp. 11649–11661, Apr. 2013.

[74] B. György, T. G. Szabó, L. Turiák, M. Wright, P. Herczeg, Z. Lédeczi, Kittel, A. Polgár, K. Tóth, B. Dérfalvi, G. Zelenák, I. Böröcz, B. Carr, G. Nagy, K. Vékey, S. Gay, A. Falus, and E. I. Buzás, "Improved Flow Cytometric Assessment Reveals Distinct Microvesicle (Cell-Derived Microparticle) Signatures in Joint Diseases," *PLoS ONE*, vol. 7, p. e49726, Nov. 2012.

[75] R. Wubbolts, R. S. Leckie, P. T. M. Veenhuizen, G. Schwarzmann, W. Möbius, J. Hoernschemeyer, J.-W. Slot, H. J. Geuze, and W. Stoorvogel, "Proteomic and biochemical analyses of human B cell-derived exosomes. Potential implications for their function and multivesicular body formation," *J Biol Chem*, vol. 278, pp. 10963–10972, Mar. 2003.

[76] Y. Senju and F.-C. Tsai, "A biophysical perspective of the regulatory mechanisms of ezrin/radixin/moesin proteins," *Biophys Rev*, vol. 14, pp. 199–208, Jan. 2022.

[77] D. B. Peterson, T. Sander, S. Kaul, B. T. Wakim, B. Halligan, S. Twigger, K. A. Pritchard, K. T. Oldham, and J.-S. Ou, "Comparative proteomic analysis of PAI-1 and TNF-alpha-derived endothelial microparticles," *Proteomics*, vol. 8, pp. 2430–2446, June 2008.

[78] J. Dalli, T. Montero-Melendez, L. V. Norling, X. Yin, C. Hinds, D. Haskard, M. Mayr, and M. Perretti, "Heterogeneity in neutrophil microparticles reveals distinct proteome and functional properties," *Mol Cell Proteomics*, vol. 12, pp. 2205–2219, Aug. 2013.

[79] A. Stadtmann and A. Zarbock, "The role of kindlin in neutrophil recruitment to inflammatory sites:," *Current Opinion in Hematology*, vol. 24, pp. 38–45, Jan. 2017.

[80] S. C. Fagerholm, H. S. Lek, and V. L. Morrison, "Kindlin-3 in the immune system," *Am J Clin Exp Immunol*, vol. 3, pp. 37–42, Feb. 2014.

[81] L. Miguet, K. Pacaud, C. Felden, B. Hugel, M. C. Martinez, J.-M. Freyssinet, R. Herbrecht, N. Potier, A. van Dorsselaer, and L. Mauvieux, "Proteomic analysis of malignant lymphocyte membrane microparticles using double ionization coverage optimization," *Proteomics*, vol. 6, pp. 153–171, Jan. 2006.

[82] S. N. Hurwitz, M. A. Rider, J. L. Bundy, X. Liu, R. K. Singh, and D. G. Meckes, "Proteomic profiling of NCI-60 extracellular vesicles uncovers common protein cargo and cancer type-specific biomarkers," *Oncotarget*, vol. 7, pp. 86999–87015, Dec. 2016.

[83] G. J. C. G. M. Bosman, E. Lasonder, M. Luten, B. Roerdinkholder-Stoelwinder, V. M. J. Novotný, H. Bos, and W. J. De Grip, "The proteome of red cell membranes and vesicles during storage in blood bank conditions," *Transfusion*, vol. 48, pp. 827–835, May 2008.

[84] B. A. Garcia, D. M. Smalley, H. Cho, J. Shabanowitz, K. Ley, and D. F. Hunt, "The platelet microparticle proteome," *J Proteome Res*, vol. 4, no. 5, pp. 1516–1521, 2005.

[85] B. Kinner and M. Spector, "Smooth muscle actin expression by human articular chondrocytes and their contraction of a collagen-glycosaminoglycan matrix in vitro," *J Orthop Res*, vol. 19, pp. 233–241, Mar. 2001.

[86] A. Ludin, T. Itkin, S. Gur-Cohen, A. Mildner, E. Shezen, K. Golan, O. Kollet, A. Kalinkovich, Z. Porat, G. D'Uva, A. Schajnovitz, E. Voronov, D. A. Brenner, R. N. Apte, S. Jung, and T. Lapidot, "Monocytes-macrophages that express -smooth muscle actin preserve primitive hematopoietic cells in the bone marrow," *Nat*

Immunol, vol. 13, pp. 1072–1082, Nov. 2012. Number: 11 Publisher: Nature Publishing Group.

[87] Z. Liu, A. N. Chang, F. Grinnell, K. M. Trybus, D. M. Milewicz, J. T. Stull, and K. E. Kamm, "Vascular disease-causing mutation, smooth muscle -actin R258C, dominantly suppresses functions of -actin in human patient fibroblasts," *Proceedings of the National Academy of Sciences*, vol. 114, pp. E5569–E5578, July 2017. Publisher: Proceedings of the National Academy of Sciences.

[88] J. Wang, R. Zohar, and C. A. McCulloch, "Multiple roles of alpha-smooth muscle actin in mechanotransduction," *Exp Cell Res*, vol. 312, pp. 205–214, Feb. 2006.

[89] H. Y. Song, M. Y. Kim, K. H. Kim, I. H. Lee, S. H. Shin, J. S. Lee, and J. H. Kim, "Synovial fluid of patients with rheumatoid arthritis induces -smooth muscle actin in human adipose tissue-derived mesenchymal stem cells through a TGF-1-dependent mechanism," *Exp Mol Med*, vol. 42, pp. 565–573, Aug. 2010.

[90] D. L. Mattey, P. T. Dawes, N. B. Nixon, and H. Slater, "Transforming growth factor beta 1 and interleukin 4 induced alpha smooth muscle actin expression and myofibroblast-like differentiation in human synovial fibroblasts in vitro: modulation by basic fibroblast growth factor," *Ann Rheum Dis*, vol. 56, pp. 426–431, July 1997.

[91] T. W. Kragstrup, D. H. Sohn, C. M. Lepus, K. Onuma, Q. Wang, W. H. Robinson, and J. Sokolove, "Fibroblast-like synovial cell production of extra domain A fibronectin associates with inflammation in osteoarthritis," *BMC Rheumatol*, vol. 3, p. 46, Dec. 2019.

[92] R. H. L. Li, N. Nguyen, T. Rosati, and K. Jandrey, "Assessment of P2Y12 Inhibition by Clopidogrel in Feline Platelets Using Flow Cytometry Quantification of Vasodilator-Stimulated Phosphoprotein Phosphorylation," *Frontiers in Veterinary Science*, vol. 7, 2020.

[93] D.-S. Choi, D.-Y. Choi, B. S. Hong, S. C. Jang, D.-K. Kim, J. Lee, Y.-K. Kim, K. P. Kim, and Y. S. Gho, "Quantitative proteomics of extracellular vesicles derived from human primary and metastatic colorectal cancer cells," *J Extracell Vesicles*, vol. 1, 2012.

[94] B. J. Tauro, R. A. Mathias, D. W. Greening, S. K. Gopal, H. Ji, E. A. Kapp, B. M. Coleman, A. F. Hill, U. Kusebauch, J. L. Hallows, D. Shteynberg, R. L. Moritz, H.-J. Zhu, and R. J. Simpson, "Oncogenic H-ras reprograms Madin-Darby canine kidney (MDCK) cell-derived exosomal proteins following epithelial-mesenchymal transition," *Mol Cell Proteomics*, vol. 12, pp. 2148–2159, Aug. 2013.

[95] H. Hu, C. Li, H. Zhang, G. Wu, and Y. Huang, "Role of vasodilator-stimulated phosphoprotein in RANKL-differentiated murine macrophage RAW264.7 cells: Modulation of NF-B, c-Fos and NFATc1 transcription factors," *Experimental and Therapeutic Medicine*, vol. 21, pp. 1–9, May 2021.

[96] S. Mii, A. Enomoto, Y. Shiraki, T. Taki, Y. Murakumo, and M. Takahashi, "CD109: a multifunctional GPI-anchored protein with key roles in tumor progression and physiological homeostasis," *Pathology International*, vol. 69, no. 5, pp. 249–259, 2019.

[97] D. M. Mallawaaratchy, S. Hallal, B. Russell, L. Ly, S. Ebrahimkhani, H. Wei, R. I. Christopherson, M. E. Buckland, and K. L. Kaufman, "Comprehensive proteome profiling of glioblastoma-derived extracellular vesicles identifies markers for more aggressive disease," *J Neurooncol*, vol. 131, pp. 233–244, Jan. 2017.

[98] G. Song, T. Feng, R. Zhao, Q. Lu, Y. Diao, Q. Guo, Z. Wang, Y. Zhang, L. Ge, J. Pan, L. Wang, and J. Han, "CD109 regulates the inflammatory response and is required for the pathogenesis of rheumatoid arthritis," *Annals of the Rheumatic Diseases*, vol. 78, pp. 1632–1641, Dec. 2019. Publisher: BMJ Publishing Group Ltd Section: Rheumatoid arthritis.

[99] X.-Y. Man, K. W. Finnson, M. Baron, and A. Philip, "CD109, a TGF- co-receptor, attenuates extracellular matrix production in scleroderma skin fibroblasts," *Arthritis Research & Therapy*, vol. 14, p. R144, June 2012.

[100] J. Yang, X.-Z. Zhan, J. Malola, Z.-Y. Li, J. S. Pawar, H.-T. Zhang, and Z.-G. Zha, "The multiple roles of Thy-1 in cell differentiation and regeneration," *Differentiation*, vol. 113, pp. 38–48, May 2020.

[101] J.-H. Schroeder, G. Beattie, J. W. Lo, T. Zabinski, N. Powell, J. F. Neves, R. G. Jenner, and G. M. Lord, "CD90 is not constitutively expressed in functional innate lymphoid cells," *Front Immunol*, vol. 14, p. 1113735, Apr. 2023.

[102] A. K. Wiles, S. Mehta, M. Millier, A. G. Woolley, K. Li, K. Parker, M. Kazantseva, M. Wilson, K. Young, S. Bowie, S. Ray, T. L. Slatter, L. K. Stamp, P. A. Hessian, and A. W. Braithwaite, "Activated CD90/Thy-1 fibroblasts co-express the 133p53 isoform and are associated with highly inflamed rheumatoid arthritis," *Arthritis Res Ther*, vol. 25, p. 62, Apr. 2023.

[103] A. Saalbach and U. Anderegg, "Thy-1: more than a marker for mesenchymal stromal cells," *The FASEB Journal*, vol. 33, no. 6, pp. 6689–6696, 2019.

[104] J.-W. Bae, J. K. Yi, E.-J. Jeong, W.-J. Lee, J.-M. Hwang, D.-H. Kim, J. J. Ha, and W.-S. Kwon, "Ras-related proteins (Rab) play significant roles in sperm motility and capacitation status," *Reproductive Biology*, vol. 22, p. 100617, June 2022.

[105] N. S. Vasileva, E. V. Kuligina, M. A. Dymova, Y. I. Savinovskaya, N. D. Zinchenko, A. B. Ageenko, S. V. Mishinov, A. S. Dome, G. A. Stepanov, V. A. Richter, and D. V. Semenov, "Transcriptome Changes in Glioma Cells Cultivated under Conditions of Neurosphere Formation," *Cells*, vol. 11, p. 3106, Oct. 2022.

[106] B. Vastrad, C. Vastrad, and A. Tengli, "Bioinformatics analyses of significant genes, related pathways, and candidate diagnostic biomarkers and molecular targets in SARS-CoV-2/COVID-19," *Gene Reports*, vol. 21, p. 100956, Dec. 2020.

[107] X.-H. Liang, Z.-P. Feng, F.-Q. Liu, R. Yan, L.-Y. Yin, H. Shen, and H.-L. Lu, "Identification of potential biomarkers for diagnosis of hepatocellular carcinoma," *Experimental and Therapeutic Medicine*, vol. 23, pp. 1–10, Jan. 2022.

[108] A. Arrazola Sastre, M. Luque Montoro, P. Gálvez-Martín, H. M. Lacerda, A. Lucia, F. Llavero, and J. L. Zugaza, "Small GTPases of the Ras and Rho Families Switch on/off Signaling Pathways in Neurodegenerative Diseases," *International Journal of Molecular Sciences*, vol. 21, p. 6312, Jan. 2020.

[109] R. V. Koziy, J. L. Bracamonte, S. Yoshimura, P. Chumala, E. Simko, and G. S. Katselis, "Discovery proteomics for the detection of putative markers for eradication of infection in an experimental model of equine septic arthritis using LC-MS/MS," *Journal of Proteomics*, vol. 261, p. 104571, June 2022.

[110] T. Hasegawa, H. Asanuma, J. Ogino, Y. Hirohashi, Y. Shinomura, H. Iwaki, H. Kikuchi, and T. Kondo, "Use of potassium channel tetramerization domain-containing 12 as a biomarker for diagnosis and prognosis of gastrointestinal stromal tumor," *Human Pathology*, vol. 44, pp. 1271–1277, July 2013.

[111] Z. Wang, D. Wu, M. Dong, Y. Xia, and T. Xu, "KCTD12 is a prognostic marker of breast cancer and correlates with tumor immune cell infiltration," *Transl Cancer Res*, vol. 10, pp. 261–272, Jan. 2021.

[112] K. Okubo, H. Wada, A. Tanaka, H. Eguchi, M. Hamaguchi, A. Tomokuni, Y. Tomimaru, T. Asaoka, N. Hama, K. Kawamoto, S. Kobayashi, S. Marubashi, H. Nagano, N. Sakaguchi, H. Nishikawa, Y. Doki, M. Mori, and S. Sakaguchi, "Identification of Novel and Noninvasive Biomarkers of Acute Cellular Rejection After Liver Transplantation by Protein Microarray," *Transplant Direct*, vol. 2, p. e118, Nov. 2016.

[113] R. Ye, X. Kuang, H. Zeng, N. Shao, Y. Lin, and S. Wang, "KCTD12 promotes G1/S transition of breast cancer cell through activating the AKT/FOXO1 signaling," *J Clin Lab Anal*, vol. 34, p. e23315, Mar. 2020.

[114] Z. Liu, Y. Xiang, and G. Sun, "The KCTD family of proteins: structure, function, disease relevance," *Cell & Bioscience*, vol. 3, p. 45, Nov. 2013.
